# Supplementary material for: GenomePAM directs PAM characterization and engineering of CRISPR-Cas nucleases using mammalian genome repeats
Source: Nat Biomed Eng. 2025 Aug 13;10(2):231–44. doi: 10.1038/s41551-025-01464-y (PMC12812445; doi:10.1038/s41551-025-01464-y)
Supplement: Supplementary file 1 — Reporting Summary [file 41551_2025_1464_MOESM1_ESM.pdf]

nature portfolio

Corresponding author(s): Zongli Zheng

Last updated by author(s): May 11, 2025

Reporting Summary

Nature Portfolio wishes to improve the reproducibility of the work that we publish. This form provides structure for consistency and transparency in reporting. For further information on Nature Portfolio policies, see our Editorial Policies and the Editorial Policy Checklist.

Statistics

For all statistical analyses, confirm that the following items are present in the figure legend, table legend, main text, or Methods section.

n/a	Confirmed	
☐	☒	The exact sample size (*n*) for each experimental group/condition, given as a discrete number and unit of measurement
☐	☒	A statement on whether measurements were taken from distinct samples or whether the same sample was measured repeatedly
☐	☒	The statistical test(s) used AND whether they are one- or two-sided<br>*Only common tests should be described solely by name; describe more complex techniques in the Methods section.*
☒	☐	A description of all covariates tested
☒	☐	A description of any assumptions or corrections, such as tests of normality and adjustment for multiple comparisons
☐	☒	A full description of the statistical parameters including central tendency (e.g. means) or other basic estimates (e.g. regression coefficient) AND variation (e.g. standard deviation) or associated estimates of uncertainty (e.g. confidence intervals)
☐	☒	For null hypothesis testing, the test statistic (e.g. *F*, *t*, *r*) with confidence intervals, effect sizes, degrees of freedom and *P* value noted<br>*Give P values as exact values whenever suitable.*
☒	☐	For Bayesian analysis, information on the choice of priors and Markov chain Monte Carlo settings
☒	☐	For hierarchical and complex designs, identification of the appropriate level for tests and full reporting of outcomes
☒	☐	Estimates of effect sizes (e.g. Cohen's *d*, Pearson's *r*), indicating how they were calculated

Our web collection on statistics for biologists contains articles on many of the points above.

Software and code

Policy information about availability of computer code

Data collection	The study developed new codes: https://github.com/Zheng-NGS-Lab/genomePAM; All datasets generated by this study have been deposited at SRA (ID 1258724 - BioProject - NCBI) (https://www.ncbi.nlm.nih.gov/bioproject/PRJNA1258724). Human genome reference hg38 was collected from UCSC (https://hgdownload.soe.ucsc.edu/goldenpath/hg38/bigZips/).
Data analysis	https://github.com/tsailabSJ/guideseq; BWA version: 0.7.17-r1188; Samtools version: 1.8 (htslib 1.8). R version 4.1.2; ggseqlogo version 0.2.

For manuscripts utilizing custom algorithms or software that are central to the research but not yet described in published literature, software must be made available to editors and reviewers. We strongly encourage code deposition in a community repository (e.g. GitHub). See the Nature Portfolio guidelines for submitting code & software for further information.

Data

Policy information about availability of data

All manuscripts must include a data availability statement. This statement should provide the following information, where applicable:

- Accession codes, unique identifiers, or web links for publicly available datasets
- A description of any restrictions on data availability
- For clinical datasets or third party data, please ensure that the statement adheres to our policy

Detail target sites identified have been included in the supplementary Excel files. Raw Fastq data will be deposit at SRA before publication.

April 2023

Research involving human participants, their data, or biological material

Policy information about studies with human participants or human data. See also policy information about sex, gender (identity/presentation), and sexual orientation and race, ethnicity and racism.

Reporting on sex and gender	N/A
Reporting on race, ethnicity, or other socially relevant groupings	N/A
Population characteristics	N/A
Recruitment	N/A
Ethics oversight	N/A

Note that full information on the approval of the study protocol must also be provided in the manuscript.

Field-specific reporting

Please select the one below that is the best fit for your research. If you are not sure, read the appropriate sections before making your selection.

☒ Life sciences ☐ Behavioural & social sciences ☐ Ecological, evolutionary & environmental sciences

For a reference copy of the document with all sections, see nature.com/documents/nr-reporting-summary-flat.pdf

Life sciences study design

All studies must disclose on these points even when the disclosure is negative.

Sample size	The is one place in our manuscript mentioning 'n', in Line 281: "large-scale synthetic oligos (n = 26,891)". This was from a previous study that we cited, to demonstrate the advantage of our study without the need to synthesis any oligo but to use sequence already in the genome.
Data exclusions	No data were excluded from the analyses
Replication	All experiments were replicated at least 3.
Randomization	The study did not involve human or animal subjects. All cell line experiments started with relevant cell lines and were replicated at least 3 times.
Blinding	The study did not involve human or animal subjects. All cell line experiments started with relevant cell lines and were replicated at least 3 times.

Reporting for specific materials, systems and methods

We require information from authors about some types of materials, experimental systems and methods used in many studies. Here, indicate whether each material, system or method listed is relevant to your study. If you are not sure if a list item applies to your research, read the appropriate section before selecting a response.

Materials & experimental systems

n/a	Involved in the study	
☒	☐	Antibodies
☐	☒	Eukaryotic cell lines
☒	☐	Palaeontology and archaeology
☒	☐	Animals and other organisms
☒	☐	Clinical data
☒	☐	Dual use research of concern
☒	☐	Plants

Methods

n/a	Involved in the study	
☒	☐	ChIP-seq
☒	☐	Flow cytometry
☒	☐	MRI-based neuroimaging

Eukaryotic cell lines

Policy information about cell lines and Sex and Gender in Research

Cell line source(s)	All cell lines were purchased from ATCC (American type culture collection). HEK293T, HepG2, Huh7 and HeLa
Authentication	All cell lines used were authenticated by STR (Short Tandem Repeat) when purchased from ATCC
Mycoplasma contamination	All cell lines were tested negative for mycoplasma contamination
Commonly misidentified lines (See ICLAC register)	No commonly misidentified cell lines were used in the study

Plants

Seed stocks	N/A
Novel plant genotypes	N/A
Authentication	N/A
